# Estimating Adult and Old Age Mortality From Parental Survival Histories Collected Over the Phone: A Validation Study From Bangladesh

**DOI:** 10.1111/tmi.70173

**Published:** 2026-06-09

**Authors:** Md. Tazvir Amin, Md. Mahabubur Rahman, M. Moinuddin Haider, Nurul Alam, Georges Reniers, Stephane Helleringer

**Affiliations:** ^1^ Health Systems and Population Studies Division Icddr,b Dhaka Bangladesh; ^2^ Global Health and Migration Unit, Department of Women's and Children's Health Uppsala University Uppsala Sweden; ^3^ Department of Population Health London School of Hygiene & Tropical Medicine London UK; ^4^ Division of Social Science, Program in Social Research and Public Policy New York University Abu Dhabi UAE

**Keywords:** adult and old age mortality, Bangladesh, mobile phone surveys, parental survival histories

## Abstract

**Objectives:**

Bangladesh, a lower‐middle‐income (LMIC) South Asian country, relies on survey data for measuring adult mortality. Mobile phone surveys are an appealing alternative to in‐person surveys because they are cheaper and can be deployed when enumerator mobility is hindered. In this contribution, we examined the quality of ‘Parental Survival History’ data collected over the phone by comparing it with data from a Health and Demographic Surveillance System.

**Methods:**

We conducted a mobile phone survey from July to December 2021 among residents of the Matlab HDSS. Mobile phone survey data about the respondent's father and mother were individually matched to the HDSS database. Data were compared to the HDSS reference in terms of the vital status of the respondent's parents, their age, the time since death and adult mortality rates.

**Results:**

A total of 2231 respondents reported information on their parents. Among these, 90.7% of fathers' names and 95.2% of mothers' names were matched between the mobile phone survey and the HDSS, and the matched dataset was used for subsequent analyses. Concordance in the vital status reports of the parent was very high (both sensitivity and specificity were above 99%). Further, we found that 50% of the parents' ages (current age for those who are alive and age at death for parents who died) were within 5 years below and 3 years above those recorded in the HDSS. Around 4% of parental deaths were erroneously located within or outside an 8‐year reference period. The parents' mortality rate was 52.0 (95% CI: 49.3, 54.8) per 1000 person‐years in the mobile phone survey and 52.3 (95% CI: 49.6, 55.0) in the HDSS.

**Conclusions:**

In this population, parental survival history data were reported with high accuracy in a mobile phone survey, highlighting their potential for measuring adult mortality in resource‐constrained settings.

## 1 Background

Global health has undergone remarkable transitions in the recent past, including a spectacular drop in child mortality in most low‐ and middle‐income countries (LMICs) [1]. As child mortality falls, an increasing proportion of deaths occur in adulthood and old age, and this will amplify in the coming decades [2, 3, 4]. In LMICs, adult mortality is attributable to HIV/AIDS, maternal mortality, external injuries and—increasingly—non‐communicable diseases and ageing‐related conditions [5, 6, 7, 8, 9, 10, 11, 12]. Estimating and monitoring age and cause‐specific adult mortality is crucial for strengthening policy formulation and resource allocation [13]. It is a public health priority for tracking and achieving the Sustainable Development Goals (SDG 3) [14, 15].

Over 100 LMICs lack functional ‘Civil Registration and Vital Statistics’ systems, and continuous population‐based data for monitoring progress is scarce [16, 17]. LMICs, therefore, rely on in‐person surveys and censuses to address this data gap, and these typically make use of modules or instruments that inquire about survival of household members or relatives (e.g., recent household deaths, sibling survival histories and parental survival histories) [13, 18, 19, 20].

In Bangladesh, sibling survival histories and recent household deaths have been used as data collection tools in surveys to collect mortality data for a long time, but parental survival history has not been used to date. The approach was first proposed by Henry in 1960 and rests on a few simple questions about the survival status of the respondent's biological parents [21, 22]. Parental survival history data are relatively easy to collect at scale and an excellent data source for adult and old age mortality estimation via direct or indirect methods [21, 23].

The rise in mobile phone ownership presents new opportunities for data collection. In addition to simpler logistics, mobile phone surveys can also be deployed during pandemics or humanitarian crises when in‐person data collection may be hindered. The experience with mobile phone survey‐based mortality estimates is, however, limited, and a comprehensive assessment of the quality of parental survival history data collected through mobile phone surveys is essential. To fill this evidence gap, a mobile phone survey was undertaken in 2021 in a subsample of Health and Demographic Surveillance System (HDSS) residents in Matlab, a rural area in Bangladesh. The objectives of this study are as follows: (a) to compare mobile phone survey‐generated parental survival history data against the reference data from the Matlab HDSS and (b) to compare the mortality rates derived from the mobile phone survey to those observed in the HDSS.

## 2 Methods

### 2.1 Design, Settings, and Participants

The icddr,b has been recording vital events in the Matlab HDSS area since 1966 [24]. About 240,000 individuals, mostly in rural communities, are covered by the HDSS [24, 25]. Most people currently residing in the Matlab HDSS have been followed from birth [24]. Those living in the HDSS before 1966 were enumerated in the initial census of the de jure population [24]. Female community interviewers conducted monthly household visits until 2006 and bi‐monthly visits thereafter to record vital events, including births, deaths, migration, marriages and marital disruptions [26]. The HDSS ensures data accuracy and completeness through a two‐tier supervision system and an independent quality control team [26]. Persons who leave the HDSS area for 6 months or longer are considered out‐migrants and no longer followed [24]. The Matlab HDSS dataset serves as the reference data against which we compared mobile phone survey data. We sampled individuals from the Matlab HDSS database using a stratified random sample design and invited them to participate in the mobile phone survey. To ensure that a sufficient number of deaths were captured for mortality estimation, we oversampled respondents with a parent who died in the past 3 years. Details of the sampling method used in this mobile phone survey study will be found in previously published work [27, 28, 29, 30]. Mobile phone survey data collection took place from July to December 2021.

### 2.2 Matching of Parents Between Surveys and HDSS

The Matlab HDSS's relational database allows users to link individuals with their relatives, including parents, via the mother ID [31]. In the mobile phone survey, respondents reported the names of their father and mother, and these were manually matched to the HDSS database for comparing data across sources. Matching was only possible for parents who also resided in the HDSS area. Cases were treated as a match provided that any portion of their name or full name corresponded in the two data sources. The information for matched parents, including vital status, was then extracted from the HDSS database. Individual names in the HDSS and mobile phone survey may differ due to variations in nicknames and the official name of an individual, variations in spelling and enumerator errors.

### 2.3 Outcome Measures

The primary outcomes of this study were reporting errors in parental information collected through the mobile phone survey, including errors in vital status, age (current age for those who are alive and age at death for parents who died) and time since death. In addition, we compared (direct) adult mortality rates estimated from parental survival history collected through the mobile phone survey with mortality rates derived from the Matlab HDSS.

Vital status errors occur when the vital status of the parent is not in agreement between the mobile phone survey and the HDSS database. We quantify the accuracy of parental survival history in accurately classifying parental vital status by calculating the sensitivity (the proportion of deceased parents in the HDSS correctly identified as such in the mobile phone survey) and specificity (the proportion of living parents correctly reported as alive in the mobile phone survey according to the Matlab HDSS database).

Age reporting errors are common in surveys, even for respondents' self‐reports [32], and we anticipated that this problem would be exacerbated for parental age (alive and deceased). To assess age reporting errors, we computed the difference between the age of parents as reported in the mobile phone survey and that in the HDSS. Positive values are indicative of age overreporting in the mobile phone survey, while negative values indicate underreporting.

Event dating errors have repercussions for the analysis of mortality trends [33]. To assess the dating errors, we categorized the time since death into two groups as follows: Those who died during an 8‐year reference period before the survey, versus parents who died earlier. We estimated the sensitivity (the fraction of HDSS deaths in the reference period reported as such in the mobile phone survey) and specificity (the fraction of HDSS deaths before the reference period reported as such in the mobile phone survey). This is followed by a more detailed analysis of the distribution of differences between the two data sources in the year of death.

Mortality rates (aged 30–95) were calculated for parents using the parental survival history data. First, a unique identification variable was created for each parent. Second, the dataset was expanded to generate person‐years covering the 8 years preceding the survey. As per convention, age‐specific mortality rates (_n_m_x_) were computed by dividing the number of deaths by person‐years at risk. We used life table methods for converting mortality rates into probabilities of dying in adulthood and old age (_n_q_x_) [34].

### 2.4 Ethics Approval and Consent to Participate

The study protocol was reviewed and approved by the Institutional Review Board (IRB) of the International Centre for Diarrhoeal Disease Research, Bangladesh (icddr,b) (IRB No.: 00001822; Federalwide Assurance [FWA] No.: FWA00001468). All participants provided informed consent prior to participation in the study, including consent for data collection, analysis, and the use of anonymized data for research and publication purposes.

## 3 Results

### 3.1 Respondent Characteristics

Out of 2565 sampled cases, 2214 (86%) interviews were completed. In addition, 17 interviews were partially completed but contained complete parental survival history information and were therefore retained for analysis. The remaining sampled cases were partially complete without usable parental survival history information (*N* = 28), refused (*N* = 32), or could not be reached (*N* = 276). Table 1 represents the distribution of socio‐demographic characteristics of the mobile phone survey respondents. The percentage of females was 13% points lower than that of males, and 22% were over 50 years old. Around 8% of respondents had no education, whereas just over 23% had higher education. The majority of respondents in the mobile phone survey identified as Muslim.

**TABLE 1:** Background characteristics of mobile phone survey respondents.

Respondent's characteristics	Mobile phone survey	
	%	*n*
Sex		
Male	56.5	1259
Female	43.5	970
Age		
15–29	34.3	765
30–39	21.8	486
40–49	21.8	485
50–83	22.1	493
Education		
None	7.9	177
Primary	29.3	654
Secondary	39.3	876
Higher	23.4	522
Religion		
Muslim	86.6	1930
Non‐Muslim	13.4	299
Total	100	2229

*Note:* Socio‐demographic background information for two respondents was missing. In the mobile phone survey, we had a strategy for taking the parental survival history information first and then completing the respondent background section later. For these particular respondents, the interview was interrupted after giving the parental survival history information.

### 3.2 Matching Parents Between the Mobile Phone Survey and the HDSS

On the HDSS end, respondents were first linked to their parents to extract their demographic data and survival status information. We successfully linked 94% of respondents to the HDSS record of their father and 98.9% of respondents to their mother in the HDSS database (Table 2). The names of fathers and mothers as recorded in the HDSS databases were thereafter compared to those reported by mobile phone survey respondents. Names did not match for 63 fathers and for 83 mothers across both sources, resulting in an analytical dataset of 2023 fathers and 2123 mothers.

**TABLE 2:** Mobile phone survey respondents and the analytical dataset.

Status	Father		Mother	
	*N*	%	*N*	%
Sampled cases	2565		2565	
Respondents with complete parental survival history	2231	100	2231	100
HDSS record with linked parent	2086	93.5	2206	98.9
Matched mobile phone survey‐HDSS records	2023	90.7	2123	95.2

*Note:* Percentage computed out of a total of 2231 parents.

### 3.3 Comparison of Parent Attributes in Matched/Unmatched Cases

Completeness in reporting the parents' current age, age at death and time since death was close to 100% in the mobile phone survey (Table 3). In comparison to cases that were matched to an HDSS record, unmatched parents were more likely to have deceased, and this is particularly pronounced for men. Unmatched cases also seem to be characterised by a younger age at death, suggesting that these events took place a longer time ago. For parents who were still alive, the median age of fathers was 7 (matched) and 5 (unmatched) years higher than for mothers. For matched cases, the median age at death for fathers and mothers was quasi‐identical.

**TABLE 3:** Parent attributes for mobile phone survey respondents with and without an HDSS match.

Parent's characteristics	Father				Mother			
	Matched		Unmatched		Matched		Unmatched	
	*N*	%	*N*	%	*N*	%	*N*	%
Vital status								
Alive	610	30.2	33	15.9	1355	63.8	65	60.2
Deceased	1412	69.8	172	82.7	768	36.2	43	39.8
Unknown	1	0.05	3	1.4	—	—	—	—
Current age reported in the mobile phone survey	610	100	33	100	1354	99.9	65	100
Age at death reported in the mobile phone survey	1392	98.6	159	92.4	751	97.8	42	97.7
Year of death in the mobile phone survey	1411	99.9	166	96.5	766	99.7	42	97.7
Age distribution (25th, 50th and 75th percentiles)								
Current age (alive)	(52, 62, 70)	—	(50, 60, 68)	—	(46, 55, 65)	—	(47, 55, 65)	—
Age at death (deceased)	(58, 69, 80)	—	(48, 58, 70)	—	(60, 70, 80)	—	(55, 65, 75)	—

*Note:* Vital status was missing for 4 fathers in the mobile phone survey. The survey did not record the ages of 17 mothers and 20 fathers.

### 3.4 Reporting Errors

Table 4 demonstrates a high concordance of vital status reports between the mobile phone survey and the HDSS. The sensitivity and specificity of the mobile phone survey using the HDSS as a reference were above 99%.

**TABLE 4:** Sensitivity and specificity of vital status reports in mobile phone survey relative to HDSS.

	HDSS record	Mobile phone survey record		
		Alive *n* %	Deceased *n* %	Total (*n*)
Father	Alive	578 99.5% (Sn)	5 0.5%	583
	Deceased	7 0.2%	1396 99.8% (Sp)	1403
Mother	Alive	1321 99.3% (Sn)	9 0.7%	1330
	Deceased	1 0.1%	752 99.9% (Sp)	753

Abbreviations: Sn, Sensitivity; Sp, Specificity.

Age reporting errors by the sex of the parent, survival status, and age are documented in Figure 1. This indicates that the variance in the age reports is a bit higher for deceased compared to alive parents.

**FIGURE 1:**
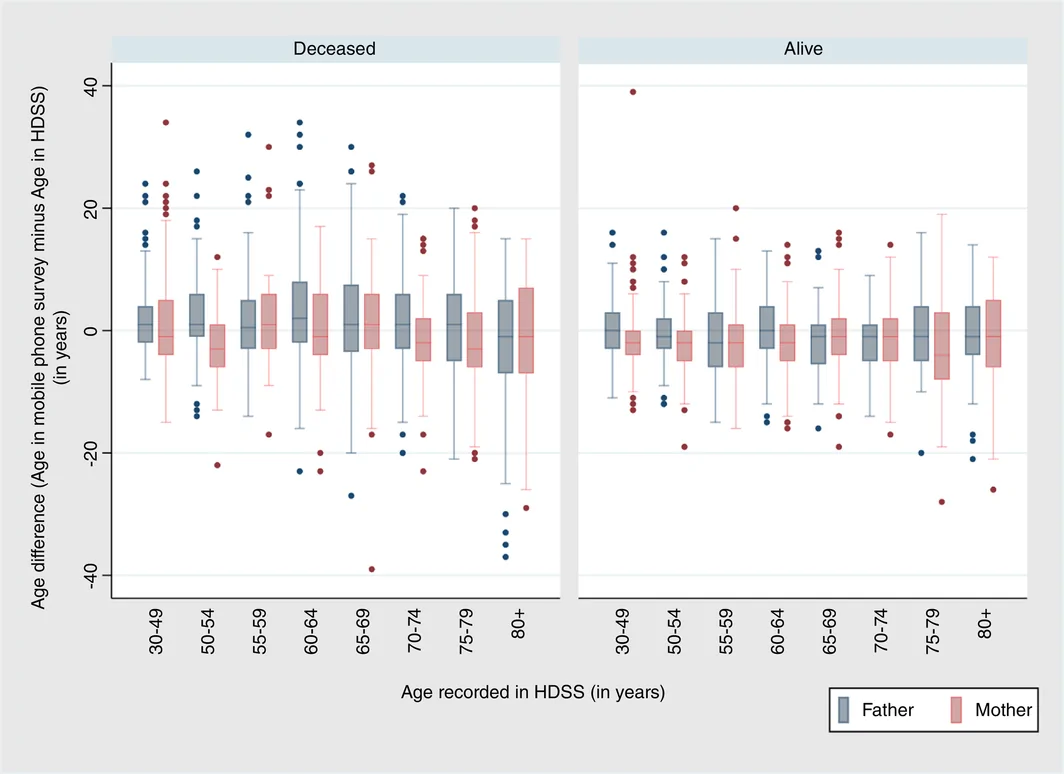
Distribution of age differences in the mobile phone survey and HDSS, by sex, survival status, and age of the parent.

The accuracy in the date of death reporting is documented in Table 5. The mobile phone survey has a sensitivity of 96.1% and a specificity of 95.7% for correctly placing a father's death in the 8‐year reference period before the survey. For mothers, the sensitivity is 96.2, and the specificity is 94.7%. Figure 2 further suggests that reporting errors in the year of death rapidly increase for deaths that occurred 10 or more years ago, and this is more pronounced for fathers than for mothers.

**TABLE 5:** Comparing the date error between HDSS and the mobile phone survey.

	HDSS record	Mobile phone survey record		
		Died in ref. period *n* %	Died before ref. period *n* (%)	Total (*n*)
Father	Died in ref. period	784 96.1% (Sn)	32 3.9%	816
	Died before ref. period	32 4.3%	554 95.7% (Sp)	579
Mother	Died in ref. period	482 96.2% (Sn)	29 3.8%	501
	Died before ref. period	13 5.3%	232 94.7% (Sp)	245

Abbreviations: ref, reference period 8 years preceding the survey; Sn, Sensitivity; Sp, Specificity.

**FIGURE 2:**
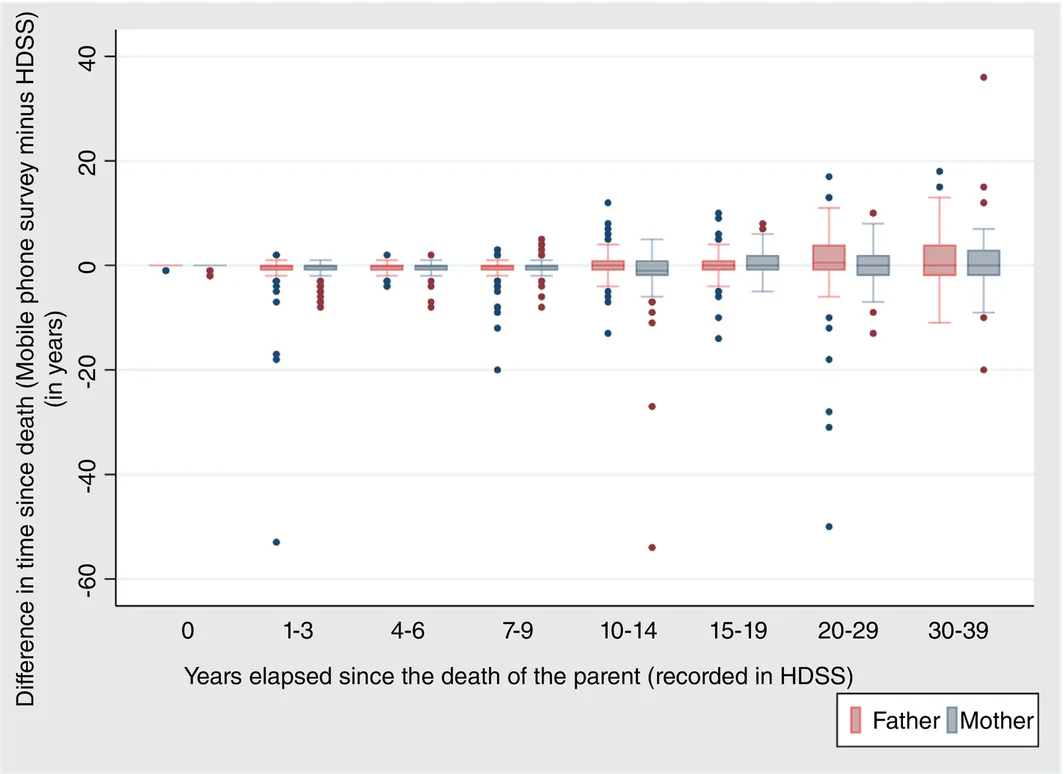
The distribution of mobile phone survey‐HDSS differences in the reported year of death, by sex of the parent, and the time since death as recorded in the HDSS.

### 3.5 Mortality Rates

Table 6 presents the age‐specific mortality rates and probabilities of dying for fathers and mothers across different age groups, comparing estimates from the mobile phone survey and HDSS. For fathers, mortality rates increase consistently with age, peaking in the 95+ age group. The overall mortality rate for fathers per 1000 person‐years was 78.6 (95% CI: 73.6–83.9) in the mobile phone survey and 78.7 (95% CI: 73.7–83.9) in HDSS, demonstrating a high degree of consistency between the two sources. As expected, the mortality rates for mothers were lower than those of fathers across all age groups. The overall mortality rate was 33.6 (95% CI: 30.8–36.5) per 1000 person‐years in the mobile phone survey and 34.0 (95% CI: 31.3–37.0) in the HDSS.

**TABLE 6:** Mortality rates and probabilities of dying for the 8 years before the survey by gender, age, and source.

Age group	Mobile phone survey				HDSS			
	Person‐years	Deaths	Mortality rate per 1000 (95% CI) (_n_m_x_)	_n_q_x_	Person‐years	Deaths	Mortality rate per 1000 (95% CI) (_n_m_x_)	_n_q_x_
Father								
30–39	437	8	18.3 [9.2, 36.2]	0.1677	250	2	8.0 [2, 31.5]	0.0769
40–44	761	18	23.7 [14.9, 37.2]	0.1117	580	18	31.0 [19.6, 48.7]	0.144
45–49	1080	41	38.0 [28.1, 51.2]	0.1734	1059	47	44.4 [33.5, 58.6]	0.1997
50–54	1240	64	51.6 [40.6, 65.4]	0.2286	1571	94	59.8 [49.1, 72.7]	0.2602
55–59	1328	101	76.1 [63, 91.6]	0.3195	1628	121	74.3 [62.5, 88.1]	0.3134
60–64	1304	86	66.0 [53.7, 80.8]	0.2831	1124	104	92.5 [76.9, 110.9]	0.3757
65–69	1219	104	85.3 [70.9, 102.4]	0.3516	1002	58	57.9 [45, 74.2]	0.2528
70–74	959	98	102.2 [84.5, 123]	0.407	1161	75	64.6 [51.8, 80.3]	0.2781
75–79	758	82	108.2 [88, 132.4]	0.4258	1034	127	122.8 [104.2, 144.3]	0.4698
80–84	591	72	121.8 [97.8, 150.8]	0.4669	745	86	115.4 [94.4, 140.5]	0.4479
85–89	438	59	134.7 [105.8, 170]	0.5038	376	66	175.5 [140.3, 217.4]	0.61
90–94	395	37	93.7 [68.6, 126.7]	0.3795	131	35	267.2 [198.2, 349.7]	0.8009
95+	74	62	837.8 [734.9, 905.9]	—	41	9	219.5 [117.4, 372.9]	—
Mother								
30–39	2186	12	5.5 [3.1, 9.6]	0.0534	1548	8	5.2 [2.6, 10.3]	0.0504
40–44	1712	15	8.8 [5.3, 14.5]	0.0429	1476	15	10.2 [6.1, 16.8]	0.0496
45–49	1766	20	11.3 [7.3, 17.5]	0.0551	1782	18	10.1 [6.4, 16]	0.0493
50–54	1600	14	8.8 [5.2, 14.7]	0.0428	1863	30	16.1 [11.3, 22.9]	0.0774
55–59	1696	21	12.4 [8.1, 18.9]	0.0601	1850	21	11.4 [7.4, 17.3]	0.0552
60–64	1746	38	21.8 [15.9, 29.8]	0.1032	1641	28	17.1 [11.8, 24.6]	0.0818
65–69	1549	53	34.2 [26.2, 44.5]	0.1576	1534	35	22.8 [16.4, 31.6]	0.1079
70–74	1056	89	84.3 [69, 102.6]	0.3481	1546	81	52.4 [42.3, 64.7]	0.2316
75–79	756	70	92.6 [73.9, 115.4]	0.3759	1212	110	90.8 [75.8, 108.3]	0.3699
80–84	435	58	133.3 [104.5, 168.7]	0.5	645	96	148.8 [123.4, 178.5]	0.5424
85–89	328	34	103.7 [75, 141.7]	0.4116	282	56	198.6 [156, 249.4]	0.6635
90–94	332	33	99.4 [71.5, 136.6]	0.3981	101	26	257.4 [181.3, 351.8]	0.7831
95+	65	54	830.8 [718.6, 904.2]	—	9	3	333.3 [103.1, 685]	—

Abbreviation: _n_q_x_, probabilities of dying.

Among fathers, mortality rates are relatively low before Age 50 but increase significantly thereafter. The probability of dying between Ages 40 and 60 (20q40) is estimated at 61.5% in the mobile phone survey and 65.2% in the HDSS. For mothers, the probability of dying between Ages 40 and 60 (20q40) was estimated at 18.6% in the mobile phone survey and 21.2% in the HDSS. As expected, mortality rates rise sharply after Age 60. The probability of dying between 60 and 80 years (20q60) reaches 84.2% for fathers. This is closely aligned with the HDSS estimate of 82.1%. For mothers, the probability of dying between 60 and 80 years of age (20q60) was 69.3% in the mobile phone survey and 60.3% in the HDSS. Uncertainty around these estimates tends to increase with age as the data become sparser.

## 4 Discussion

Mobile phone surveys have emerged as a plausible alternative to in‐person surveys during epidemic outbreaks, conflict situations, and other humanitarian emergencies and can also be used to assess mortality [18]. Via a comparison with high‐quality, prospectively collected HDSS data, this study underscored the viability of adult mortality estimation from parental survival history data collected via a mobile phone survey.

We found near 100% accuracy in mobile phone survey reporting of parents' vital status (sensitivity > 99%, specificity > 99%). The finding was expected because individuals in Matlab are generally aware of their parents' vital status. Many children live with parents in the same household or close to the parental household and regularly communicate with their parents [35, 36]. Thus, misreporting of parental survival status is highly unlikely, except for data entry errors and deliberate misreporting. There is no socio‐cultural and religious taboo against reporting a parental death either, and this study demonstrates that respondents are also willing to share this information in a mobile phone survey.

We also observed high concordance between the mobile phone survey and HDSS in the placement of parental deaths within the 8‐year reference period preceding the survey, with sensitivities of 96.1% for fathers and 96.2% for mothers, and specificities of 95.7% and 94.7%, respectively. About 99% of the population in Bangladesh is affiliated with Islam and Hinduism [37]. More generally, reporting of the year of death is very good provided that the death occurred in the last decade, and deteriorates thereafter. In Bangladesh, it is common to observe yearly remembrance for deceased parents [38]. This helps offspring and relatives recall these dates and times since death. Deterioration in the accuracy with which older events are reported will matter less for mortality estimates from parental survival history, as these are typically restricted to a 5‐ or 10‐year reference period prior to the survey.

Ages of parents, whether alive or deceased, are reported with greater error, but we could not identify any systematic upwards or downward bias in the mobile phone survey data. Further, these errors will be less pertinent for summary measures of mortality over a broader age range (e.g., 20q40 or 20q60). These summary measures are indicative of a close correspondence between the mobile phone survey estimates and those directly derived from prospective surveillance data from the Matlab HDSS.

## 5 Limitations

This study is very encouraging in the sense that it demonstrates high correspondence between mobile phone survey and HDSS data for data points that are necessary for estimating adult and old‐age mortality. This suggests that mobile phone surveys are a viable modality for collecting parental survival history and adult mortality estimates. This conclusion comes, however, with several caveats. First, this validation study was conducted in a population repeatedly exposed to research and with whom Matlab researchers established long‐lasting relationships. Among others, this resulted in atypically high response rates for the mobile phone survey. Second, the analyses are restricted to a subgroup of parents who also live(d) in the Matlab HDSS area and therefore live(d) in proximity to the respondents. It may well be that reporting accuracy is worse for parents who live(d) further away. Similarly, the analysis is restricted to parents who could be matched on their names, and it is plausible that discrepancies in the reporting of names are correlated with reporting errors on ages and dates.

## 6 Conclusion

This study demonstrates the potential of mobile phone surveys to yield reliable parental survival history data. High and increasing ownership of mobile phones in LMICs [39] makes mobile phone surveys a cost‐effective and operationally feasible alternative for monitoring adult and old‐age mortality in settings where civil registration and vital statistics systems remain incomplete.

## Funding

This study was made possible with financial support from the Bill and Melinda Gates Foundation (INV‐023211). The funder had no role in study design, data collection, data analysis, data interpretation or write‐up. Core support for icddr,b and the Matlab HDSS comes from the Governments of the People's Republic of Bangladesh and Canada.

## Conflicts of Interest

The authors declare no conflicts of interest.

## Data Availability

The data used in this study can be requested from Dr. Nurul Alam (Principal Investigator, icddr,b nalam@icddrb.org).
